# Immunosuppressive-Therapy Management and Day-14 Molecular Remission in Ulcerative Colitis Complicated by Cytomegalovirus Colitis: An Exploratory Prospective Cohort Study

**DOI:** 10.3390/medicina62081532

**Published:** 2026-08-09

**Authors:** Zeki Islamoglu, Hasan Yilmaz, Ali Erkan Duman, Goktug Sirin, Murat Sayan

**Affiliations:** 1 Department of Gastroenterology, University of Health Sciences Kocaeli City Hospital, 41285 Izmit, Kocaeli, Turkey; 2 Department of Gastroenterology, Research and Training Hospital, Kocaeli University, 41380 Izmit, Kocaeli, Turkey; gyrusus@yahoo.com (H.Y.); dralierkanduman@hotmail.com (A.E.D.); gsirin@live.com (G.S.); 3 PCR Unit, Clinical Laboratory, Research and Training Hospital, Kocaeli University, 41380 Izmit, Kocaeli, Turkey; sayanmurat@gmail.com; 4 DESAM Research Institute, Near East University, 99138 Nicosia, North Cyprus, Turkey

**Keywords:** ulcerative colitis, cytomegalovirus colitis, immunosuppressive agents, molecular remission, ganciclovir

## Abstract

*Background and Objectives:* Evidence regarding continuation versus withdrawal of immunosuppressive agents during antiviral treatment of cytomegalovirus (CMV) colitis complicating ulcerative colitis (UC) remains limited. We compared molecular remission, clinical outcomes, and relapse according to immunosuppressive-therapy management. *Materials and Methods:* This prospective, non-randomized, single-center cohort included 44 hospitalized adults with UC and CMV colitis treated with intravenous ganciclovir. Patients were managed according to a clinician-selected strategy of temporary discontinuation (*n* = 24) or continuation (*n* = 20) of immunosuppressive therapy. Molecular remission, the primary outcome, was defined as undetectable tissue CMV DNA at day 14. *Results:* Both groups demonstrated significant clinical and endoscopic improvement. The continuation group had higher residual CMV DNA levels at day 14 (*p* = 0.003) and more adverse events (*p* = 0.019). Clinical remission, endoscopic remission, and one-year relapse rates were comparable. Multivariable logistic regression showed that continuation of immunosuppressive therapy was associated with reduced molecular remission (OR 0.26, 95% CI 0.07–0.98; *p* = 0.045). Corticosteroid exposure was confined to the continuation group (7/20 vs. 0/24), raising the possibility of substantial residual confounding. *Conclusions:* In this exploratory cohort, clinician-selected continuation of immunosuppressive therapy was associated with a lower likelihood of day-14 molecular remission and more frequent adverse events, without demonstrable additional clinical or endoscopic benefit. However, the observed association may have been influenced by corticosteroid exposure and should not be interpreted as a class-wide effect of immunosuppressive therapy. These hypothesis-generating findings require confirmation in adequately powered multicenter studies before informing routine clinical practice.

## 1. Introduction

Ulcerative colitis (UC) is a chronic inflammatory bowel disease characterized by mucosal inflammation of the colon and rectum [1,2]. Cytomegalovirus (CMV) infection frequently complicates UC, particularly in patients with non-mild disease who require immunosuppressive therapies [3]. CMV, a member of the Herpesviridae family, establishes lifelong latency after primary infection and may reactivate under conditions of immunosuppression, leading to colonic inflammation [4]. CMV prevalence is around 16–34% in moderate-to-severe UC flares, increasing up to 43–67% in steroid-refractory cases [5,6]. Reactivation is associated with treatment resistance, increased colectomy rates, and prolonged hospital stays, yet the exact pathogenic role of CMV is debated [7].

Early studies focused on prevalence and treatment impacts [3,5]; however, diagnosis of CMV infection was largely reliant upon serology or qualitative PCR results that do not factor in viral load. Systematic reviews also report diagnostic challenges with serological markers, quantitative PCR (qPCR), and immunohistochemistry [7,8]. CMV positivity has been associated with steroid resistance in patients with IBD, although heterogeneity in diagnostic methods and uncertainty regarding the direction of causality complicate the interpretation of this association [6]. Current ECCO guidance states that immunosuppressive therapy can generally be continued in patients with intestinal CMV reactivation, whereas corticosteroid tapering is advised and treatment interruption is reserved for symptomatic disseminated CMV infection [9]. Nevertheless, evidence regarding continuation versus withdrawal of immunosuppressive therapy during antiviral treatment remains limited. Few studies have evaluated the impact of this management strategy on clinically relevant outcomes such as molecular remission, viral clearance, and relapse rates [10]. Additionally, the role of newer agents such as anti-tumor necrosis factor (TNF) therapies, JAK inhibitors, and anti-integrins in CMV reactivation remains unclear because viral complications are often underreported or excluded from clinical trials [11].

Uncertainty regarding the optimal management of immunosuppressive therapy during CMV colitis may lead to suboptimal treatment decisions and increased morbidity, particularly in patients with refractory UC [12]. Therefore, this prospective cohort study aimed to compare molecular remission, clinical outcomes, and relapse rates between patients who continued immunosuppressive therapy and those in whom it was temporarily discontinued during antiviral treatment for CMV colitis complicating UC.

## 2. Materials and Methods

### 2.1. Study Setting and Ethics

This prospective non-randomized cohort study was conducted at the Gastroenterology Department of Kocaeli University Research and Application Hospital in Kocaeli, Turkey, between June 2018 and June 2024. Ethical approval was obtained from the Kocaeli University Non-Interventional Clinical Research Ethics Committee (Date: 5 June 2018, No: 2018/257). The study was conducted in accordance with the principles of the Declaration of Helsinki. Written informed consent was obtained from all participants. The study was reported in accordance with the Strengthening the Reporting of Observational Studies in Epidemiology (STROBE) statement.

### 2.2. Participant Enrollment and Study Design

The study population consisted of consecutive adult patients with active UC who developed concomitant CMV colitis while receiving immunosuppressive therapy and required inpatient treatment. UC was diagnosed according to established clinical, endoscopic, and histopathological criteria. CMV colitis was diagnosed by detection of CMV DNA using polymerase chain reaction (PCR) in colonic tissue samples obtained from patients with compatible clinical and endoscopic findings.

At the time of CMV colitis diagnosis, all patients in the immunosuppression-discontinued group (24/24) were receiving azathioprine as part of their UC treatment. In this group, azathioprine was temporarily withheld during ganciclovir therapy. In the immunosuppression-continued group, ongoing immunosuppressive treatment was maintained during antiviral therapy; 13 of 20 patients continued azathioprine. Other concomitant immunosuppressive agents used in the continued group are shown in Table 1.

Following confirmation of CMV colitis, all patients received intravenous ganciclovir therapy and were hospitalized for a standardized two-week treatment period. Patients were classified according to whether immunosuppressive therapy was temporarily discontinued or continued during antiviral treatment. The decision was made by the treating gastroenterologist according to routine clinical practice based on disease severity, previous treatment history, clinical response, and individual risk–benefit considerations. No predefined study protocol was used to determine treatment allocation. Accordingly, because the continuation group included heterogeneous and potentially overlapping immunosuppressive regimens, the exposure evaluated in this study should be interpreted as a clinician-selected immunosuppressive-management strategy rather than as a homogeneous pharmacological class effect. Because CMV colitis is a relatively uncommon complication of UC, no formal sample size calculation was performed; all eligible patients during the study period were included.

Inclusion criteria were age ≥ 18 years, diagnosis of UC with concomitant CMV colitis, and receipt of optimized immunosuppressive treatment before CMV diagnosis. Exclusion criteria included contraindication to ganciclovir therapy, refusal to provide informed consent, previous total colectomy, pregnancy, lactation, active malignancy, uncontrolled non-CMV infection, or severe hepatic dysfunction.

### 2.3. Data Collection and Clinical Assessments

Data were collected prospectively through clinical assessments, laboratory investigations, endoscopic examinations, and patient-reported questionnaires using standardized data collection forms. In line with the increasing use of innovative non-invasive assessment modalities in prospective clinical research [13], standardized protocols were applied throughout the study period. Baseline assessments were performed at admission, with follow-up evaluations on days 7 and 14. Disease severity was assessed using the Truelove and Witts Severity Index [14]. Endoscopic disease activity was assessed using the Mayo Endoscopic Score [15] and the Ulcerative Colitis Endoscopic Index of Severity (UCEIS) [16]. Clinical disease activity was evaluated using the full Mayo Disease Activity Index (Mayo DAI) [17], which ranges from 0 to 12 points. Clinical remission was defined as a total Mayo score ≤ 2 with no individual subscore > 1. Clinical response was defined as a decrease of at least 3 points from baseline. Colonic biopsy specimens underwent manual digestion with Proteinase K, followed by automated DNA extraction using QIAGEN EZ1 magnetic-particle technology and the EZ1&2 DNA Tissue Kit (QIAGEN GmbH, Hilden, Germany). According to the manufacturer’s protocol, the accepted solid-tissue input range was 10–40 mg. Tissue CMV DNA was quantified at baseline and day 14 using the Artus QIAGEN CMV DNA Kit (QIAGEN GmbH, Hilden, Germany). Quantitative results were generated by the laboratory and reported as copies per millilitre of the final DNA eluate. Because the exact weight of each biopsy specimen and the total extracted DNA concentration were not recorded, the results could not be normalized to tissue weight or total DNA mass.

### 2.4. Study Outcomes

The primary outcome was molecular remission at day 14, defined as undetectable tissue CMV DNA by quantitative PCR. Secondary outcomes included changes in tissue CMV DNA load, Mayo DAI, Mayo Endoscopic Score, UCEIS, clinical response, clinical remission, endoscopic remission, treatment-related adverse events, and relapse within one year.

Three virological outcomes were distinguished. Molecular remission (primary outcome) was defined as an undetectable tissue CMV-DNA result at day 14. A study-defined robust virological response (secondary exploratory outcome) was defined as either a ≥2-log_10_ (≥100-fold) reduction in tissue CMV DNA from baseline or conversion to an undetectable result at day 14. Because no validated response threshold exists for serial tissue CMV-DNA measurements in CMV colitis, this stringent criterion was chosen to capture a marked antiviral effect. A less stringent threshold (≥1-log_10_ reduction or undetectable conversion) was evaluated as a sensitivity analysis. ‘Viral clearance’ is used descriptively in the Discussion to refer to the overall reduction in tissue CMV-DNA burden and is not a separately defined outcome measure.

### 2.5. Statistical Analysis

All statistical analyses were performed using IBM SPSS Statistics version 27.0 (IBM Corp., Armonk, NY, USA). A two-sided *p*-value < 0.05 was considered statistically significant. Distribution normality was assessed using the Shapiro–Wilk test. Continuous variables were presented as mean ± standard deviation or median (25th–75th percentile). Between-group comparisons were performed using Student’s *t*-test or Mann–Whitney U test. Repeated measurements were analyzed using two-way repeated-measures ANOVA or Friedman/Wilcoxon signed-rank tests with Bonferroni correction. Categorical variables were compared using the chi-square test, Fisher’s exact test, or Fisher–Freeman–Halton test as appropriate. No missing data were present for the primary outcome analysis. Because a substantial proportion of day-14 tissue CMV-DNA values were below the limit of detection and could not be log-transformed directly, the baseline-adjusted comparison of day-14 viral load between groups was performed using Quade’s non-parametric rank-analysis-of-covariance procedure.

Variables for the multivariable logistic regression model were selected based on clinical relevance and a priori knowledge. Age, baseline tissue CMV DNA load (log_10_-transformed), and baseline UCEIS score were included as covariates. The number of candidate predictors was constrained by the events per variable (EPV) rule, as the study included 21 molecular remission events, yielding an EPV of 5.25 for a four-variable model. Model performance was evaluated using the Hosmer–Lemeshow goodness-of-fit test, the concordance statistic (C-statistic), and variance inflation factors (VIF). The E-value was calculated according to VanderWeele and Ding [18]. As a sensitivity analysis, baseline lymphocyte count was added to the primary model.

## 3. Results

**Figure 1:**
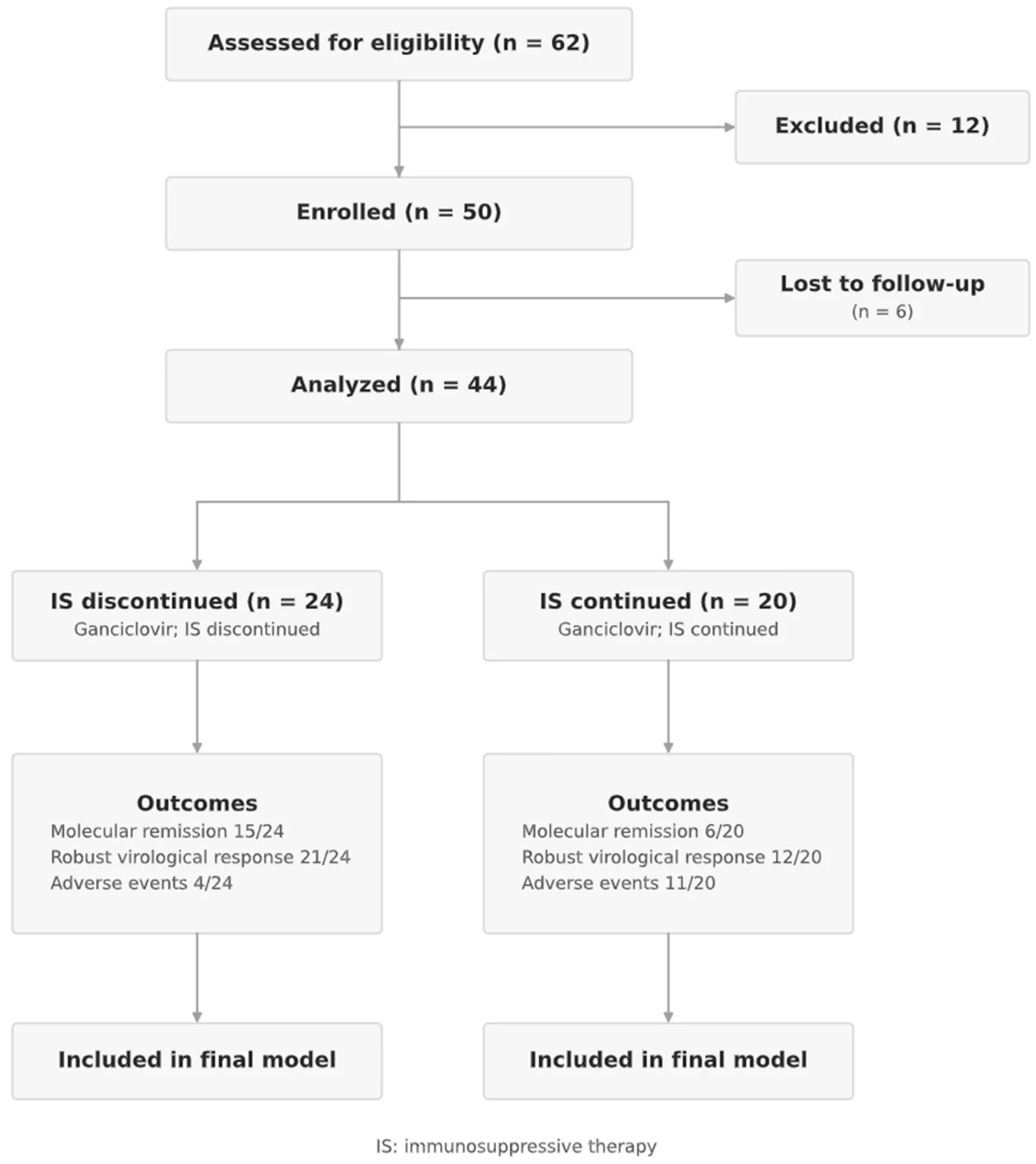
STROBE flow diagram of patient enrollment and study analysis. IS: immunosuppressive therapy.

A total of 62 patients were assessed for eligibility. After exclusion of 12 patients, 50 eligible patients were enrolled. Six patients were lost to follow-up, leaving 44 patients for the final analysis: immunosuppressive therapy was discontinued in 24 and continued in 20 patients (Figure 1).

The overall median age was 53 years (range 36–63). The study population consisted predominantly of males (70.5%, *n* = 31). Baseline demographic, clinical, laboratory, and endoscopic characteristics were comparable between groups (Table 1). Adverse events were significantly more common among those continuing immunosuppression (55.0%) than in the immunosuppressive-free group (16.7%) (*p* = 0.019). Hypotension and vertigo were observed exclusively in those continuing immunosuppressive treatment (*p* = 0.014).

During ganciclovir therapy, 13 of 20 patients in the continued group remained on azathioprine, whereas all immunosuppressive agents were withheld in the discontinued group.

Both groups showed significant improvement in daily stool frequency and bloody stool frequency from baseline to day 14 (*p* < 0.001 within each group). Tissue CMV DNA load decreased significantly in both groups; however, at day 14, the continued-immunosuppressive group had significantly higher residual CMV DNA load (*p* = 0.003). Robust virological response was numerically higher in the discontinued group (87.5% vs. 60.0%), although this difference did not reach statistical significance (*p* = 0.078). In a sensitivity analysis using a ≥1-log_10_ reduction from baseline or conversion to an undetectable result, virological response was observed in 24 of 24 patients (100%) in the discontinued group and 13 of 20 patients (65.0%) in the continued group (Fisher’s exact *p* = 0.002). Rates of endoscopic remission, clinical response, clinical remission, and relapse within one year were comparable (Table 2).

Laboratory measurements showed baseline lymphocyte counts were significantly lower in the continued group (*p* = 0.038), persisting at day 7 (*p* = 0.007) (Table S3).

Multivariable logistic regression analysis identified 21 patients (47.7%) who achieved molecular remission (15/24 in the discontinued group vs. 6/20 in the continued group). Continuation of immunosuppressive therapy was associated with a lower likelihood of achieving molecular remission (OR: 0.26, 95% CI: 0.07–0.98, *p* = 0.045), after adjustment for age, baseline CMV DNA load (log_10_-transformed), and baseline UCEIS score (Table 3).

Because baseline tissue CMV-DNA load was numerically higher in the continued group, we performed a baseline-adjusted comparison of day-14 viral load using Quade’s non-parametric rank-analysis-of-covariance procedure. After adjustment for baseline rank CMV-DNA load, patients continuing immunosuppressive therapy still had significantly higher day-14 CMV DNA than those discontinuing therapy (adjusted rank difference 10.1, 95% CI 3.3–16.9; *p* = 0.005), indicating that the observed group difference was not attributable to baseline viral-load imbalance alone.

The model demonstrated acceptable goodness-of-fit (Hosmer–Lemeshow: *p* = 0.349) and moderate discriminative ability (C-statistic = 0.729). All VIF values were below 1.2. The E-value for the point estimate was 7.15, indicating that an unmeasured confounder would need at least 7-fold association to explain the result. However, the E-value for the CI bound was 1.16.

In a sensitivity analysis, baseline lymphocyte count was added to the primary model. The association remained virtually unchanged (OR: 0.25, 95% CI: 0.06–0.97, *p* = 0.046), and lymphocyte count was not independently associated with the outcome (OR: 0.91, *p* = 0.820; Table 4).

## 4. Discussion

Our study suggests an association whereby concurrent immunosuppressive therapy during antiviral treatment for CMV colitis in UC patients is linked to lower molecular remission and increased recorded adverse events, while clinical symptom improvement and endoscopic healing remain comparable. Specifically, patients continuing immunosuppressive therapy exhibited higher residual tissue CMV DNA loads at day 14, a numerically lower rate of robust virological response that did not reach statistical significance, and more frequent adverse events—particularly hypotension and vertigo. Despite these differences, both groups demonstrated significant reductions in stool frequency, bloody stools, and inflammatory markers, with no significant between-group variations in endoscopic remission or relapse rates within one year. Multivariable logistic regression demonstrated that continuation of immunosuppressive therapy was associated with reduced molecular remission (OR 0.26, 95% CI 0.07–0.98; *p* = 0.045), after adjustment for age, baseline CMV DNA load, and baseline disease severity.

It should be noted that among the 20 patients who continued immunosuppressive therapy, 7 (35%) were receiving corticosteroids, which directly promote CMV replication through glucocorticoid response elements in the CMV genome. Among steroid users in the continued group, only 1 of 7 (14.3%) achieved molecular remission, compared with 5 of 13 (38.5%) non-corticosteroid immunosuppressive users. This difference did not reach statistical significance (Fisher’s exact test, OR 0.27, 95% CI 0.02–2.53; *p* = 0.354), likely reflecting limited statistical power given the small subgroup sizes. Although the sample size precludes definitive subgroup analysis, the observed association may have been partly or substantially influenced by corticosteroid exposure rather than representing a uniform effect of immunosuppressive therapy. Due to EPV constraints (5.25 with 4 predictors), inclusion of additional covariates was not feasible. Future studies with larger sample sizes should address whether corticosteroids versus non-steroidal agents differentially affect viral clearance.

An important finding is the dissociation between clinical/endoscopic improvement and virological clearance. Despite comparable clinical and endoscopic responses, patients who continued immunosuppressive therapy had significantly higher residual tissue CMV DNA and a numerically lower rate of robust virological response that did not reach statistical significance. This suggests that standard response criteria may not fully capture ongoing viral activity, and reliance solely on symptomatic improvement may underestimate persistent CMV replication [19,20].

We selected day-14 molecular remission as the primary endpoint because persistent tissue CMV DNA may represent ongoing low-grade viral replication that precedes, rather than accompanies, macroscopic clinical or endoscopic deterioration; a purely clinical/endoscopic endpoint could therefore underestimate incomplete viral eradication even when symptomatic improvement is already evident. Although undetectable tissue CMV DNA was selected as the primary endpoint, the clinical implications of residual low-level viral DNA remain incompletely defined. One-year relapse rates were similar, and we were not powered to detect long-term outcome differences. Nevertheless, molecular endpoints were selected because tissue CMV DNA provides an objective and reproducible measure of antiviral efficacy [8], persistent CMV DNA has been associated with steroid refractoriness and increased colectomy risk [21], and complete viral clearance represents the most conservative virological target [22]. Recent evidence suggests that achieving concurrent endoscopic and histologic remission is associated with favorable long-term prognosis [23], in contrast to pediatric data where CMV infection was associated with higher colectomy rates [24]. Future studies are needed to establish whether molecular remission translates into improved long-term outcomes.

CMV complicates UC management, particularly in moderate-to-severe cases [1,3]. Our finding that continued immunosuppression was associated with lower molecular remission, together with the numerically lower remission rate among corticosteroid users, is consistent with Shukla et al., who identified corticosteroids and thiopurines, but not TNF antagonists, as risk factors for CMV reactivation [12], and with Kim et al., who similarly reported reduced ganciclovir efficacy when immunosuppression was maintained [5]. Our data extend these observations by providing a quantitative, PCR-based measure of this association rather than relying on clinical reactivation rates alone. Thiopurines suppress natural killer cell function while steroids induce viral replication via genomic mechanisms [7,21]. Our data showed comparable endoscopic remission but reduced complications with adequate antivirals (≥14 days) [25], consistent with aggregated evidence [6]. Adverse events were more frequent in the continued group (55% vs. 16.7%), including hypotension and vertigo, potentially related to the combined effects of antiviral and immunosuppressive therapies [26,27]. This is consistent with the diagnostic framework proposed by Beswick et al., who emphasized qPCR-based confirmation of pathogenic CMV over serology alone [27]. Whether biologic agents carry the same risk as corticosteroids and thiopurines remains uncertain; Mourad et al. similarly noted that the impact of biologics on CMV reactivation may differ from that of conventional immunosuppressants, a distinction our cohort was not designed to address [28]. Testing for CMV infection in treatment-refractory patients and initiating antivirals remain important factors in patient management, as CMV remains an underrated target in IBD treatment [11,29]. Diagnostic challenges in differentiating CMV colitis from IBD flares have been well documented [30].

This study has several limitations. The non-randomized design may introduce confounding by indication: although multivariable adjustment was performed and a baseline-adjusted rank-ANCOVA confirmed that the group difference in day-14 viral load persisted independent of baseline viral burden, the E-value for the confidence interval limit was only 1.16, meaning a fairly modest unmeasured confounder could still shift the estimate toward the null, and propensity score matching was not feasible given the sample size (*n* = 44). Residual confounding related to corticosteroid exposure and treatment selection is a particular concern. The continuation group included heterogeneous and potentially overlapping immunosuppressive regimens, with corticosteroid exposure confined to this group; therefore, the observed association may have been partly or substantially influenced by corticosteroid exposure and other treatment-selection factors rather than reflecting a uniform effect of immunosuppressive therapy. Accordingly, these findings should not be generalized across immunosuppressive drug classes. No a priori sample size calculation was performed, as all eligible patients during the study period were included, so the events-per-variable ratio of 5.25 falls below the conventional threshold of 10 and additional covariates could not be incorporated; the multivariable model should therefore be regarded as exploratory and hypothesis-generating rather than confirmatory. Adverse events were not graded using a standardized severity scale such as CTCAE. CMV diagnosis relied exclusively on quantitative tissue PCR, without systematic histopathological examination or immunohistochemistry. Individual biopsy weights and total extracted DNA concentrations were also not recorded, so CMV-DNA results could not be normalized to tissue weight or DNA mass; variation in tissue input, cellularity, and extraction efficiency may have influenced the absolute concentrations reported, an issue that is more relevant to the virological-response endpoint than to the primary endpoint of undetectable CMV DNA, which is less sensitive to the reporting denominator. Similarly, pre-admission corticosteroid-dependency status was not systematically recorded, so a stratified baseline comparison by this variable was not possible. Six of the 50 enrolled patients (12%) were lost to follow-up; although no data were missing for the primary outcome, selective attrition may have affected the long-term endpoints. Finally, the single-center design may limit the generalizability of these findings.

## 5. Conclusions

Continued immunosuppressive therapy during antiviral treatment for CMV colitis in patients with UC was associated with reduced molecular remission and increased adverse events after multivariable adjustment, without providing additional clinical or endoscopic benefit. However, these findings should be interpreted cautiously given the modest sample size, borderline statistical significance, and potential residual confounding, particularly by corticosteroid use. The observed association may have been influenced by corticosteroid exposure and should not be interpreted as a class-wide effect of immunosuppressive therapy. These findings are hypothesis-generating and require confirmation in adequately powered multicenter studies before informing routine clinical practice. Quantitative assessment of tissue CMV DNA may help identify persistent viral activity despite clinical improvement and may support individualized treatment monitoring in this patient population.

## Figures and Tables

**Table 1:** Summary of demographics, colitis and treatment characteristics.

Characteristic	IS Discontinued (*n* = 24)	IS Continued (*n* = 20)	*p*
Age, median year (range)	54.5 (35.5–62)	51.5 (38–64)	0.897
Gender: Male	16 (66.7%)	15 (75.0%)	0.786
Body mass index, kg/m^2^	24.0 ± 3.9	23.4 ± 2.7	0.569
Duration of disease, years	2 (1.5–5)	3.5 (2–6.5)	0.262
Comorbidity	7 (29.2%)	10 (50.0%)	0.270
Oral mesalazine use	21 (87.5%)	18 (90.0%)	1.000
Azathioprine	24 (100%)	13 (65.0%)	–
Corticosteroid	0 (0.0%)	7 (35.0%)	–
Anti-TNF	0 (0.0%)	4 (20.0%)	–
Tacrolimus	0 (0.0%)	1 (5.0%)	–
Mycophenolate mofetil	0 (0.0%)	1 (5.0%)	–
Anti-integrin	0 (0.0%)	1 (5.0%)	–
Anti-interleukin-12/23 inhibitor	0 (0.0%)	1 (5.0%)	-
TW Severity: Moderate	18 (75.0%)	13 (65.0%)	0.550
Adverse events	4 (16.7%)	11 (55.0%)	**0.019**
Hypotension	0 (0.0%)	5 (25.0%)	**0.014**
Vertigo	0 (0.0%)	5 (25.0%)	**0.014**

Immunosuppressive-agent values represent treatment exposure at the time of CMV colitis diagnosis. In the discontinued group, all listed immunosuppressive therapy was subsequently withheld during antiviral treatment, whereas the listed agents were maintained in the continued group. Agent categories were not mutually exclusive. Statistically significant *p*-values shown in bold. IS: immunosuppressive; Anti-TNF: anti-tumor necrosis factor; TW: Truelove and Witts. Complete table with all variables available in Supplementary Materials.

**Table 2:** Summary of colitis-related findings.

	IS Discontinued (*n* = 24)	IS Continued (*n* = 20)	*p*
Tissue CMV DNA, baseline (×10^3^ copies/mL of DNA eluate)	21.25 (4.70–57.55)	78.25 (3.46–329.50)	0.229
Tissue CMV DNA, day 14 (×10^3^ copies/mL of DNA eluate)	0 (0–0.07)	0.34 (0–12.45)	**0.003**
Molecular remission at day 14 (primary outcome)	15 (62.5%)	6 (30.0%)	**0.040**
Robust virological response (secondary outcome)	21 (87.5%)	12 (60.0%)	0.078
Mayo endoscopic score, baseline	3 (2.5–3)	3 (3–3)	0.193
Mayo endoscopic score, day 14	2 (2–3)	3 (2.5–3)	0.098
UCEIS, baseline	7 (5–8)	6 (5–7)	0.398
UCEIS, day 14	4.5 (4–6)	5 (3.5–6)	0.943
Mayo DAI, baseline	8 (6.5–9.5)	9 (8–10)	0.069
Mayo DAI, day 14	5.5 (4–7)	5.5 (4–8.5)	0.668
Clinical remission	1 (4.2%)	4 (20.0%)	0.160
Relapse within one year	4 (16.7%)	5 (25.0%)	0.710

Molecular remission was defined as undetectable tissue CMV DNA at day 14. Robust virological response was defined as a ≥2-log_10_ reduction from baseline or conversion to an undetectable result. Median (25th–75th percentile) for continuous, frequency (%) for categorical variables. Statistically significant *p*-values shown in bold.

**Table 3:** Multivariable logistic regression for predictors of molecular remission at day 14.

Variable	OR	95% CI	*p*	VIF
IS continuation	0.26	0.07–0.98	**0.045**	1.07
Age	0.99	0.95–1.04	0.676	1.06
Baseline CMV DNA (log_10_)	0.72	0.35–1.47	0.364	1.09
Baseline UCEIS	0.85	0.55–1.31	0.451	1.11

Hosmer–Lemeshow: χ^2^ = 6.70, *p* = 0.349; C-statistic = 0.729; McFadden R^2^ = 0.114. Total events: 21/44 (47.7%); EPV = 5.25. E-value: 7.15 (point estimate), 1.16 (CI limit). IS: immunosuppressive therapy. Statistically significant *p*-values shown in bold.

**Table 4:** Sensitivity analysis: logistic regression with baseline lymphocyte count.

Variable	OR	95% CI	*p*	VIF
IS continuation	0.25	0.06–0.97	**0.046**	1.07
Age	0.99	0.95–1.04	0.658	1.06
Baseline CMV DNA (log_10_)	0.72	0.35–1.48	0.376	1.09
Baseline UCEIS	0.85	0.55–1.32	0.468	1.13
Lymphocyte (×10^3^/µL)	0.91	0.40–2.06	0.820	1.06

EPV = 4.2 (5 predictors). IS: immunosuppressive therapy. Statistically significant *p*-values shown in bold.

## Data Availability

The data presented in this study are available on request from the corresponding author. The data are not publicly available due to privacy and ethical restrictions.

## Supplementary Materials

The following supporting information can be downloaded at: https://www.mdpi.com/article/10.3390/medicina62081532/s1, Table S1: Complete summary of demographics, colitis and treatment characteristics with regard to immunosuppressive agent use; Table S2: Complete summary of colitis-related findings with regard to immunosuppressive agent use; Table S3: Complete summary of laboratory measurements with regard to immunosuppressive agent use.

## Abbreviations

The following abbreviations are used in this manuscript:

UC	Ulcerative Colitis
CMV	Cytomegalovirus
IS	Immunosuppressive
PCR	Polymerase Chain Reaction
qPCR	Quantitative Polymerase Chain Reaction
UCEIS	Ulcerative Colitis Endoscopic Index of Severity
Mayo DAI	Mayo Disease Activity Index
TNF	Tumor Necrosis Factor
VIF	Variance Inflation Factor
EPV	Events Per Variable
OR	Odds Ratio
CI	Confidence Interval
CRP	C-Reactive Protein
CTCAE	Common Terminology Criteria for Adverse Events
STROBE	Strengthening the Reporting of Observational Studies in Epidemiology
