# Supplementary material for: Immunosuppressive-Therapy Management and Day-14 Molecular Remission in Ulcerative Colitis Complicated by Cytomegalovirus Colitis: An Exploratory Prospective Cohort Study
Source: Medicina (Kaunas). 2026 Aug 9;62(8):1532. doi: 10.3390/medicina62081532 (PMC13515517; doi:10.3390/medicina62081532)
Supplement: Supplementary file 1 [file medicina-62-01532-s001.zip › medicina-4394283-supplementary.pdf]

Supplementary Materials

Immunosuppressive-Therapy Management and Day-14 Molecular Remission in Ulcerative Colitis Complicated by Cytomegalovirus Colitis: An Exploratory Prospective Cohort Study

Table S1. Complete summary of demographics, colitis and treatment characteristics with regard to immunosuppressive agent use.

Characteristic	IS Discontinued (n = 24)	IS Continued (n = 20)	p
Age, median year (range)	54.5 (35.5–62)	51.5 (38–64)	0.897
Gender: Female	8 (33.3%)	5 (25.0%)	0.786
Gender: Male	16 (66.7%)	15 (75.0%)	
Weight, kg	68.6 ± 13.4	68.5 ± 11.3	0.983
Height, median cm (range)	170 (162–173.5)	173 (168–177)	0.277
Body mass index, kg/m²	24.0 ± 3.9	23.4 ± 2.7	0.569
Smoking: Non-smoker	14 (58.3%)	8 (40.0%)	0.436
Smoking: Ex-smoker	8 (33.3%)	9 (45.0%)	
Smoking: Active smoker	2 (8.3%)	3 (15.0%)	
Smoking pack-year	0 (0–15)	7.25 (0–30)	0.221
Alcohol: Never	19 (79.2%)	15 (75.0%)	0.121
Alcohol: Quit	2 (8.33%)	5 (25.0%)	
Alcohol: Using	3 (12.5%)	0 (0.0%)	
Age at diagnosis, median year	50 (33.5–59.5)	46 (31.5–58)	0.832
Duration of disease, years	2 (1.5–5)	3.5 (2–6.5)	0.262
Comorbidity	7 (29.2%)	10 (50.0%)	0.270
Topical mesalazine use	11 (45.8%)	7 (35.0%)	0.675
Oral mesalazine use	21 (87.5%)	18 (90.0%)	1.000
Oral mesalazine dose, mg/day	3200 (3000–4000)	3200 (3000–4000)	0.875
Azathioprine	24 (100%)	13 (65.0%)	–
Corticosteroid	0 (0.0%)	7 (35.0%)	–
Anti-TNF	0 (0.0%)	4 (20.0%)	–
Tacrolimus	0 (0.0%)	1 (5.0%)	–
Mycophenolate mofetil	0 (0.0%)	1 (5.0%)	–
Anti-integrin	0 (0.0%)	1 (5.0%)	–
Anti-interleukin-12/23 inhibitor	0 (0.0%)	1 (5.0%)	–
Abdominal surgery	6 (25.0%)	7 (35.0%)	0.695
TW Severity: Mild	5 (20.8%)	4 (20.0%)	0.550
TW Severity: Moderate	18 (75.0%)	13 (65.0%)	
TW Severity: Severe	1 (4.2%)	3 (15.0%)	
CMV IgM: Negative	16 (76.2%)	15 (75.0%)	1.000
CMV IgM: Positive	5 (23.8%)	5 (25.0%)	

CMV IgG: Positive	21 (100%)	20 (100%)	–
Colitis: Proctitis	2 (8.3%)	2 (10.0%)	0.876
Colitis: Proctosigmoiditis	4 (16.7%)	1 (5.0%)	
Colitis: Left-sided	6 (25.0%)	5 (25.0%)	
Colitis: Extensive	1 (4.2%)	1 (5.0%)	
Colitis: Pancolitis	11 (45.8%)	11 (55.0%)	
Highest ulcer diameter, mm	10 (4–20)	13.5 (10–15)	0.254
Adverse events	4 (16.7%)	11 (55.0%)	**0.019**
Headache	2 (8.3%)	5 (25.0%)	0.217
Nausea	2 (8.3%)	4 (20.0%)	0.387
Hypotension	0 (0.0%)	5 (25.0%)	**0.014**
Irritability	1 (4.2%)	1 (5.0%)	1.000
Chest pain	1 (4.2%)	0 (0.0%)	1.000
Fatigue	1 (4.2%)	2 (10.0%)	0.583
Itchy rashes	0 (0.0%)	1 (5.0%)	0.455
Leg pain	0 (0.0%)	2 (10.0%)	0.201
Vertigo	0 (0.0%)	5 (25.0%)	**0.014**
Palpitation	0 (0.0%)	1 (5.0%)	0.455

Descriptive statistics: mean ± SD for normally distributed, median (25th–75th percentile) for non-normally distributed continuous variables, frequency (%) for categorical. Statistically significant p-values shown in bold. IS: immunosuppressive; Anti-TNF: anti-tumor necrosis factor; CMV: cytomegalovirus; TW: Truelove and Witts.

Table S2. Complete summary of colitis-related findings with regard to immunosuppressive agent use.

	IS Discontinued (n = 24)	IS Continued (n = 20)	p
**Stool frequency, daily**			
Baseline	6.5 (3.5–13.5)	8 (6–10.5)	0.813
7th day	5.5 (2.5–7.5)	5 (3–7)	0.972
14th day	3 (2–5.5)	3.5 (2–6.5)	0.793
p (within groups)	<0.001	<0.001	
Change	-4.5 (-8.5 to -1)	-3 (-5.5 to -1)	0.731
**Bloody stool, daily**			
Baseline	1 (0–5)	4 (1.5–6.5)	0.099
7th day	0 (0–2)	0 (0–4.5)	0.497
14th day	0 (0–1)	0 (0–1)	0.747
p (within groups)	<0.001	<0.001	
Change	-0.5 (-2.5 to 0)	-2.5 (-4.5 to 0)	0.219
**Tissue CMV DNA (×10^3 copies/mL of DNA eluate)**			
Baseline	21.25 (4.70–57.55)	78.25 (3.46–329.50)	0.229
14th day	0 (0–0.07)	0.34 (0–12.45)	**0.003**
p (within groups)	<0.001	0.002	
Change	-21.25 (-56.91 to -4.67)	-78.04 (-302.43 to -2.71)	0.396
**Mayo endoscopic score**			
Baseline	3 (2.5–3)	3 (3–3)	0.193
14th day	2 (2–3)	3 (2.5–3)	0.098
p (within groups)	0.002	0.056	
**UCEIS**			
Baseline	7 (5–8)	6 (5–7)	0.398
14th day	4.5 (4–6)	5 (3.5–6)	0.943
p (within groups)	0.010	0.010	
**Mayo DAI**			
Baseline	8 (6.5–9.5)	9 (8–10)	0.069
14th day	5.5 (4–7)	5.5 (4–8.5)	0.668
p (within groups)	<0.001	<0.001	
Endoscopic remission, Mayo	2 (8.3%)	4 (20.0%)	0.387
Endoscopic remission, UCEIS	1 (4.2%)	4 (20.0%)	0.160
Clinical response, Mayo	10 (41.7%)	12 (60.0%)	0.364
Clinical remission, Mayo	1 (4.2%)	4 (20.0%)	0.160
Molecular remission at day 14 (primary outcome)	15 (62.5%)	6 (30.0%)	**0.040**

Robust virological response (secondary outcome)	21 (87.5%)	12 (60.0%)	0.078
Relapse within one year	4 (16.7%)	5 (25.0%)	0.710
Time to relapse, months	6 (4.5–12)	4 (3–5)	0.266

Median (25th–75th percentile) for continuous, frequency (%) for categorical variables. Change = day 14 minus baseline. Statistically significant p-values shown in bold. CMV: cytomegalovirus; DAI: disease activity index; PCR: polymerase chain reaction; UCEIS: ulcerative colitis endoscopic index of severity. Molecular remission was defined as undetectable tissue CMV DNA at day 14. Robust virological response was defined as a ≥2-$\log_{10}$ reduction from baseline or conversion to an undetectable result.

Table S3. Complete summary of laboratory measurements with regard to immunosuppressive agent use.

	IS Discontinued (n = 24)	IS Continued (n = 20)	p
**CRP, mg/L**			
Baseline	1.27 (0.63–4.99)	1.04 (0.29–3.47)	0.479
7th day	0.90 (0.44–2.51)	0.76 (0.23–1.47)	0.390
14th day	0.97 (0.50–1.84)	0.61 (0.12–1.79)	0.379
p (within groups)	0.066	0.012	
**Sedimentation, mm/h**			
Baseline	18 (12–45)	28 (9.5–35)	0.850
14th day	13.5 (9.5–39)	21 (12–35)	0.539
p (within groups)	0.214	0.819	
**Creatinine, mg/dL**			
Baseline	0.74 ± 0.21	0.69 ± 0.26	0.862
14th day	0.68 ± 0.19	0.62 ± 0.24	0.995
p (within groups)	0.049	0.202	
**Leukocyte, 10^3/μL**			
Baseline	7.64 (5.52–8.62)	7.80 (5.40–10.07)	0.916
14th day	5.82 (4.45–7.20)	5.24 (4.44–9.51)	0.714
p (within groups)	<0.001	0.016	
**Neutrophil, 10^3/μL**			
Baseline	4.15 (3.41–6.33)	5.15 (3.21–7.60)	0.532
14th day	3.49 (2.69–4.64)	3.76 (3.00–5.50)	0.174
p (within groups)	0.015	0.229	
**Lymphocyte, 10^3/μL**			
Baseline	1.77 (1.26–2.28)	1.20 (0.87–1.73)	**0.038**
7th day	1.80 (1.40–2.30)	1.03 (0.75–1.79)	**0.007**
14th day	1.55 (1.25–1.93)	0.97 (0.78–2.10)	0.089
p (within groups)	0.170	0.331	
**Platelet, 10^3/μL**			
Baseline	266.5 (191.5–385.3)	314.5 (267.5–372.5)	0.187
14th day	258.65 (207.4–305.0)	310.0 (272.0–374.0)	0.219
p (within groups)	0.142	0.623	
**MPV, fL**			
Baseline	8.20 (7.35–8.87)	7.62 (7.00–8.21)	0.083
14th day	8.00 (7.53–9.05)	7.33 (6.84–8.40)	0.154
p (within groups)	0.802	0.150	
**Albumin, g/dL**			
Baseline	3.49 (2.79–4.02)	3.43 (3.15–3.89)	0.768

14th day	3.48 (2.89–3.80)	3.50 (2.94–3.75)	0.977
p (within groups)	0.277	0.362	
**Globulin, g/dL**			
Baseline	3.01 ± 0.41	3.00 ± 0.51	0.713
14th day	2.93 ± 0.55	3.04 ± 0.60	0.472
p (within groups)	0.244	0.655	

Mean ± SD for normally distributed, median (25th–75th percentile) for non-normally distributed continuous variables. Statistically significant p-values shown in bold. CRP: C-reactive protein; MPV: mean platelet volume.
